# Supplementary material for: Maternal Factors and Their Association with Patterns of Beverage Intake in Mexican Children and Adolescents
Source: Children (Basel). 2021 May 13;8(5):385. doi: 10.3390/children8050385 (PMC8152280; doi:10.3390/children8050385)
Supplement: Supplementary file 1 [file children-08-00385-s001.zip › children-1174804-supplementary.pdf]

Table S1: Types of consumed beverages disaggregated by age- and sex-groups.

Beverages sources of TWI (ml/d)	Total sample				4 - 8 y Female				4 - 8 y Male				9 - 13 y Female				9 - 13 y Male				14 - 18 y Female				14 - 18 y Male			
	Mean	Median	IQR		Mean	Median	IQR		Mean	Median	IQR		Mean	Median	IQR		Mean	Median	IQR		Mean	Median	IQR		Mean	Median	IQR	
TWI	1771	1650	1260	2100	1511	1415	1060	1850	1699	1560	1250	2000	1768	1693	1305	2100	1831	1750	1355	2200	1812	1750	1375	2215	2032	1970	1500	2420
1. Plain water	743	600	250	1000	547	500	250	750	683	560	250	1000	791	628	250	1100	783	750	250	1175	830	750	275	1250	834	750	250	1250
Water	743	600	250	1000	547	500	250	750	683	560	250	1000	791	628	250	1100	783	750	250	1175	830	750	275	1250	834	750	250	1250
2. Tea & coffe	7	0	0	0	7	0	0	0	4	0	0	0	10	0	0	0	4	0	0	0	13	0	0	0	7	0	0	0
Tea/coffe	7	0	0	0	7	0	0	0	4	0	0	0	10	0	0	0	4	0	0	0	13	0	0	0	7	0	0	0
3. Low-fat & skim milk	71	0	0	0	68	0	0	0	77	0	0	0	72	0	0	0	63	0	0	0	71	0	0	0	73	0	0	0
Light milk	20	0	0	0	14	0	0	0	16	0	0	0	33	0	0	0	17	0	0	0	25	0	0	0	16	0	0	0
Semi skim milk	19	0	0	0	23	0	0	0	23	0	0	0	12	0	0	0	19	0	0	0	21	0	0	0	18	0	0	0
Skim milk	20	0	0	0	15	0	0	0	22	0	0	0	15	0	0	0	21	0	0	0	16	0	0	0	28	0	0	0
Low fat drinkable yogurt	1	0	0	0	2	0	0	0	1	0	0	0	1	0	0	0	1	0	0	0	0	0	0	0	0	0	0	0
Low fat yogurt	0	0	0	0	0	0	0	0	0	0	0	0	1	0	0	0	1	0	0	0	0	0	0	0	0	0	0	0
Vegetal beverages	10	0	0	0	14	0	0	0	13	0	0	0	11	0	0	0	4	0	0	0	10	0	0	0	11	0	0	0
4. Non-calorically sweetened beverages	37	0	0	0	27	0	0	0	42	0	0	0	21	0	0	0	43	0	0	0	47	0	0	0	43	0	0	0
Infusions with non-calorically sweetened	2	0	0	0	0	0	0	0	1	0	0	0	3	0	0	0	1	0	0	0	6	0	0	0	1	0	0	0
Flavored water with-non-calorically sweetened	20	0	0	0	9	0	0	0	25	0	0	0	9	0	0	0	25	0	0	0	24	0	0	0	24	0	0	0
Soft drinks with non-calorically sweetened	1	0	0	0	1	0	0	0	0	0	0	0	1	0	0	0	3	0	0	0	0	0	0	0	0	0	0	0
Industrialized drinks with non-calorically sweetened	15	0	0	0	17	0	0	0	17	0	0	0	8	0	0	0	14	0	0	0	16	0	0	0	18	0	0	0
5. Caloric beverages with some nutrients	774	735	423	1050	748	628	450	1000	794	750	430	1060	736	655	365	1035	790	750	500	1060	714	610	318	1015	860	750	500	1100
Whole milk	295	250	0	500	291	250	0	500	324	300	0	500	289	250	0	500	294	250	0	500	267	250	0	500	297	250	0	500
Flavored milk	33	0	0	0	39	0	0	0	30	0	0	0	22	0	0	0	46	0	0	0	37	0	0	0	22	0	0	0
Sugary drinkable yogurt	24	0	0	0	14	0	0	0	22	0	0	0	23	0	0	0	25	0	0	0	36	0	0	0	30	0	0	0
Sugary yogurt	12	0	0	0	13	0	0	0	14	0	0	0	13	0	0	0	10	0	0	0	10	0	0	0	11	0	0	0
Sweetened probiotic milk beverage fermented	7	0	0	0	10	0	0	0	10	0	0	0	5	0	0	0	5	0	0	0	6	0	0	0	4	0	0	0
Natural juices	14	0	0	0	10	0	0	0	8	0	0	0	12	0	0	0	16	0	0	0	31	0	0	0	13	0	0	0
Sports drinks	2	0	0	0	3	0	0	0	0	0	0	0	0	0	0	0	2	0	0	0	3	0	0	0	5	0	0	0
Fruit water	288	0	0	500	279	123	0	500	291	0	0	500	288	0	0	500	283	0	0	500	231	0	0	500	358	0	0	500
Industrialized prepared water	14	0	0	0	10	0	0	0	20	0	0	0	14	0	0	0	12	0	0	0	8	0	0	0	16	0	0	0
Industrialized juices	24	0	0	0	23	0	0	0	20	0	0	0	12	0	0	0	28	0	0	0	29	0	0	0	33	0	0	0
Nectar	55	0	0	0	50	0	0	0	45	0	0	0	55	0	0	0	62	0	0	0	53	0	0	0	66	0	0	0
Atole	7	0	0	0	7	0	0	0	10	0	0	0	4	0	0	0	9	0	0	0	5	0	0	0	5	0	0	0
6. Calorically sweetened beverages	138	0	0	250	113	0	0	250	97	0	0	120	136	0	0	250	145	0	0	250	136	0	0	250	214	0	0	290
Soda soft drinks	97	0	0	0	77	0	0	0	59	0	0	0	103	0	0	125	108	0	0	125	67	0	0	0	176	0	0	250
Frappe drinks	1	0	0	0	0	0	0	0	0	0	0	0	0	0	0	0	3	0	0	0	1	0	0	0	0	0	0	0
Jelly	6	0	0	0	9	0	0	0	9	0	0	0	7	0	0	0	2	0	0	0	5	0	0	0	3	0	0	0
Energy drinks	0	0	0	0	1	0	0	0	0	0	0	0	0	0	0	0	0	0	0	0	0	0	0	0	0	0	0	0
Sweetened infusions	48	0	0	0	34	0	0	0	53	0	0	0	35	0	0	0	35	0	0	0	101	0	0	250	52	0	0	0
Alcoholic drinks	1	0	0	0	0	0	0	0	0	0	0	0	0	0	0	0	2	0	0	0	0	0	0	0	1	0	0	0

Table S2: Relationship between maternal factors.

	MEL	SES	BPW	Mother age
MEL	–	0.545**	0.284**	0.231**
SES	0.545**	–	0.263**	0.083*
BPW	0.284**	0.263**	–	0.069**
Mother age (years)	0.231**	0.083*	0.069**	–
Mother BMI (kg/m2)	-0.093**	-0.137**	0.001	0.171**

** P value < 0.01; * P value < 0.05. Abbreviations; MEL: mothers' educational level, SES: Socioeconomic status, BPW: belongingness to the paid workforce, BMI: body mass index.
